# Trends in Nutrition and Exercise Counseling among Adolescents in the Health Care Environment

**DOI:** 10.1155/2012/949303

**Published:** 2012-08-09

**Authors:** Tasha Peart, Patricia B. Crawford

**Affiliations:** College of Natural Resources and the School of Public Health, Dr. Robert C. and Veronica Atkins Center for Weight and Health, University of California, Berkeley, CA 94704, USA

## Abstract

*Purpose*. Obesity is a serious health threat, particularly among racial/ethnic minorities and those who are uninsured, yet little is known about the implementation of nutrition or exercise counseling or the combination of both among these groups. Trends in counseling by race/ethnicity and types of insurance were examined. *Methods*. Trend analyses were conducted with the California Health Interview Surveys among those ages 12–17 for the period 2003–2009. *Results*. *Race/Ethnicity*: Receipt of both counseling methods declined from 2003–2009 for all racial/ethnic groups, except Hispanics and Whites, for whom increases in counseling began after 2007. Hispanics and African Americans generally reported higher levels of nutrition than exercise counseling, while Whites generally reported higher levels of exercise than nutrition counseling for the study period. *Insurance Type*: Receipt of both counseling methods appeared to decline from 2003–2009 among all insurance types, although after 2007, a slight increase was observed for the low-cost/free insurance group. Those with private health insurance generally received more exercise counseling than nutrition counseling over the study period. *Conclusions*. Counseling among all racial/ethnic groups and insurance types is warranted, but particularly needed for African Americans, American Indian/Alaska Natives, and the uninsured as they are at highest risk for developing obesity. Institutional and policy changes in the health care environment will be beneficial in helping to promote obesity-related counseling.

## 1. Introduction

The prevalence of obesity among adolescents in the United States has increased dramatically in the past 30 years [1], with particularly high rates among Hispanic, African American, and Native American youth [1–4]. Currently 33.6% of all adolescents 12–19 years of age in the United States are either overweight or obese [2]. Although the causes of obesity are complex, it is widely recognized that poor nutrition and physical inactivity play important roles [5]. For this reason, public health interventions targeting youths frequently focus on health promotion programs in schools [6–9], as well as calling for nutrition and exercise counseling in the health care setting [10–12]. 

The potential for physicians to influence behavioral changes among patients through simple nutrition and exercise advice, as opposed to more time-intensive counseling is crucial. Some studies have documented the value of physician-counseling either used as a stand-alone strategy [13] or as part of a coordinated effort to help patients make changes in their diet and physical activity patterns [14]. National health organizations have also recognized the importance of clinician counseling and have called for an increase in the provision of both nutrition and exercise counseling given to adolescents during physician visits [15, 16]. The Surgeon General's recent Vision for a Healthy and Fit Nation (2010) encourages clinicians to recommend healthy eating and increased physical activity to their patients and recommends training for clinicians and health care students on effective ways to counsel patients on lifestyle behavior change [17]. 

In California, where adolescent overweight and obesity rates are comparable to national levels [18], major programmatic and policy responses aimed at obesity prevention in adolescents have been undertaken at schools to limit nonnutritious foods and beverages sold on campus and in communities to increase access to nutritious foods and safer places to exercise [8, 9, 19]. However, little is known about the frequency of weight-related counseling given to different racial/ethnic groups or those with limited access to care, over the course of treating or preventing obesity in the health care environment. A recent study [20] found that race was an important factor that explained the prevalence of nutrition or physical activity counseling among California adolescents: African Americans compared to Whites were more likely to receive nutrition counseling, while Hispanics compared to Whites were more likely to receive both nutrition and physical activity counseling. With regards to insurance type, data suggests that California adolescents who are uninsured or who qualify for low-cost/free insurance are at greatest risk for overweight or obesity [21], yet a recent national study found that adolescents with private insurance generally receive more counseling, compared to those who have low-cost insurance [22]. Changes in the frequency of obesity-related counseling overtime by race/ethnicity or insurance type are yet to be examined. 

Previous research in California suggests that physician obesity-related counseling has been declining, with overall counseling rates between 2003 and 2007, shifting from 75% to 59% for nutrition and 74% to 60% for physical activity [20]. Insufficient time, lack of resources, inadequate reimbursement, and patient noncompliance are typically cited as barriers to provision of routine advice by health care practitioners [23].

The objectives of this current study were to document trends from 2003 to 2009 in either nutrition or exercise counseling or a combination of both among California adolescents by race/ethnicity and by insurance type. These findings can provide guidance for policies and programs in a state with high rates of adolescent overweight or obesity and large ethnic populations at particular risk. Further, we hypothesized that physicians would favor dietary counseling over exercise counseling when providing counseling. In an earlier study, Stafford et al. [24] found that physicians offered dietary counseling to obese patients 41.5% of the time, while exercise counseling was offered only 32.8% of the time [24]. Among healthy weight participants, Branner and colleagues found higher rates of nutrition compared with exercise counseling among children and adolescents (42.1% and 26.1%, resp.) [22].

## 2. Materials and Methods

Data demonstrating trends in nutrition and exercise counseling by race/ethnicity and by insurance type were obtained using four biennial California Health Interview Surveys (2003–2009), the largest state surveys in the United States. The California Health Interview Survey (CHIS) is a two stage sampling, weighted, random digital dialing telephone survey, representative of the California noninstitutionalized population. Within households, an adult and adolescent were randomly selected and interviewed by trained CHIS interviewers; adolescents were directly interviewed. The CHIS program obtained informed consent from all individuals participating in the survey and this current study was deemed exempt or waived for human subjects review by the University of California, Berkeley, Institutional Review Board.

## 3. Measures

### 3.1. Obesity-Related Counseling

In this study, obesity-related counseling refers to simple advice about nutrition and/or exercise practices, as opposed to more time-intensive counseling. Adolescents self-reported whether they discussed nutrition or exercise habits with their physician at their last routine exam: “When you had your last routine physical exam, did you and a doctor talk about nutrition or healthy eating?” and “When you had your last routine physical exam, did you and a doctor talk about exercise or physical activity?” 

### 3.2. Statistical Analyses

Data were analyzed using STATA version 10, with the “svy” module to account for weighting and the raking method in variance estimation. Obesity-related counseling proportions are presented graphically by race/ethnicity and by insurance type for the period 2003–2009. To better represent the obesity-related counseling construct, we categorized this variable as respondents having no discussions of nutrition or exercise with their physician, discussing either nutrition or exercise, or discussing both nutrition and exercise with their physician. Participants' self-reported their weight and height, which were used to generate the Centers for Disease Control and Prevention (CDC) BMI age and gender specific percentiles, categorized into underweight (<5th percentile), normal weight (5th–<85th percentile), overweight (85th–<95th percentile), and obese (≥95th percentile). The CDC recommends the use of BMI percentiles when assessing children's weight status [25]. In addition, insurance type variables (uninsured, Medicaid, Healthy Families, employer-based, privately owned insurance, and other public insurance) were collapsed into the categories (uninsured, low-cost/free, employer-based, and private insurance). Medicaid is the United States health insurance program for certain low-income individuals and families, which is jointly funded by state and federal governments [26], while Healthy Families is a low-cost health insurance program for children and adolescents who do not have health insurance and who do not qualify for Medicaid [27].

## 4. Results

### 4.1. Characteristics of the Study Sample (CHIS, 2009)

Table 1 presents the study sample characteristics, using the CHIS (2009). Participants ranged in age from 12 to 17 years, with 51.0% being male and 49.0% being female. The sample consisted primarily of Hispanics (49.3%) and non-Hispanic whites (33.5%). Most adolescents had some form of health insurance, with almost 60% being covered through their parents'/guardians' employer-sponsored health insurance. Less than half of all adolescents (44.8%) were at or above 300% of the federal poverty level. Based on self-reported data, 28.7% of California adolescents were either overweight or obese, while 48.2% of all California adolescents received counseling on both nutrition and exercise subjects (Table 1). The majority of respondents (84.7%) reported having a physical exam within the past year (Table 1). Previously published data indicate how California adolescent demographics have changed from 2003 to 2007 [20].

### 4.2. Either Nutrition or Exercise Counseling

**Figure 1:**
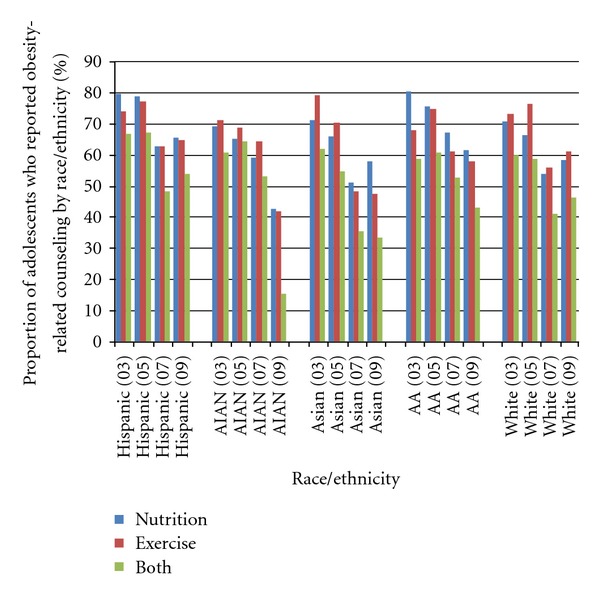
Trends in obesity-related counseling by race/ethnicity (CHIS, 2003–2009).

Figure 1 and Table 2 present data for obesity-related counseling stratified by race/ethnicity. When examining nutrition or exercise counseling separately for the period from 2003 to 2009, African Americans generally reported higher levels of nutrition than exercise counseling, while Whites generally reported higher levels of exercise than nutrition counseling. Hispanics generally reported higher levels of nutrition than exercise counseling during 2003–2005, after which counseling levels remained consistent. 

### 4.3. Both Counseling Methods 

Overall, trends show that counseling declined between 2003 and 2009 for all groups, except for Hispanics and Whites which started to increase again after 2007; American Indians/Alaska Natives reported a sharp decline in 2009.

Between 2003 and 2009, the proportion of adolescents who reported counseling on both nutrition and exercise decreased from 66.8% to 53.7% among Hispanics; from 60.7% to 15.1% among American Indians/Alaska Natives; from 61.7% to 33.4% among Asians; from 58.8% to 42.9% among African Americans; and from 60.0% to 46.2% among Whites (Figure 1). 

### 4.4. Either Nutrition or Exercise Counseling 

**Figure 2:**
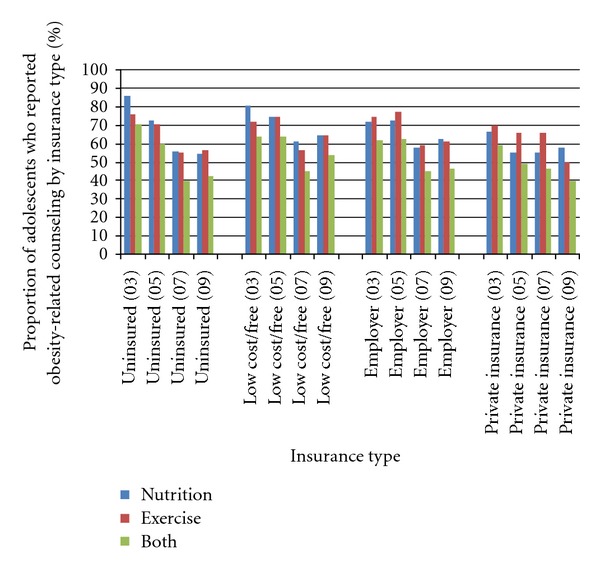
Trends in obesity-related counseling by insurance type (CHIS, 2003–2009).

Figure 2 and Table 3 present data for obesity-related counseling stratified by insurance type. Those who had private insurance generally received exercise counseling more frequently than nutrition counseling over the study time period. There appeared to be no imbalance in frequency of nutrition or exercise counseling for the uninsured, low-cost/free or employer-based groups, except in 2003 when adolescents who were underinsured or had low-cost insurance reported more nutrition than physical activity counseling.

### 4.5. Both Counseling Methods

Counseling appeared to decline from 2003–2009 among all insurance types, although after 2007, a slight increase was observed for the low-cost/free insurance group. Among those who were uninsured counseling declined from 70.8% to 42.2%; among the low-cost/free group, counseling declined from 64% to 53.4%; among the employer group, counseling declined from 61.8% to 46.6% and among the private insurance group, counseling declined from 58.9% to 39.8% (Figure 2). 

## 5. Discussion 

As early as the 1950s, the American Medical Association Council on Food and Nutrition cited the benefits of nutrition counseling, as well as inadequacies in nutrition education in U.S. medical schools [28]. Further, counseling has been shown to be valuable in helping patients to change their behavior and to achieve weight loss and can be even more beneficial if used as part of a coordinated approach with health education materials. Kreuter and colleagues reported that patients who received a combination of health education materials, followed by physician counseling were 51% more likely to increase their leisure time physical activity, and 35% more likely to reduce fat from dairy sources at follow-up [14].

Some groups that need obesity prevention counseling the most may still be missing out, including American Indians/Alaska Natives, African Americans, and the uninsured. Time trend findings from 2003 to 2009 indicate that nutrition and exercise counseling decreased for all racial/ethnic groups except for Hispanics and Whites, for whom it started to increase after 2007. 

Our findings also suggest that counseling levels in California for racial/ethnic groups and for patients with different types of insurance are generally higher compared to national levels [22]. The higher counseling levels reported in this study, compared with national figures, may have been due to fewer barriers or more public health awareness of the obesity epidemic in California and stronger health care leadership. Previously reported barriers to counseling include insufficient reimbursement rates, lack of time, lack of training for medical providers in policy advocacy related to improved nutrition and activity environments, and the need for information on evidence-based obesity-related messages and referral networks for nutrition counseling [23].

When examining nutrition or exercise counseling separately during the study period (2003–2009), interesting differences were found by race/ethnicity: Hispanic and African American CHIS adolescents generally reported higher levels of nutrition than exercise counseling, while whites generally reported higher levels of exercise, than nutrition counseling. Meanwhile, participants who had private insurance generally received more exercise than nutrition counseling during the study period. Further research is needed to investigate the underlying factors that may explain the differences in the findings for racial/ethnic groups.

## 6. Limitations

The CHIS surveys were only able to identify the existence of discussions or conversations that adolescents may have had with their physicians regarding nutrition and exercise messages, but were not able to ascertain the depth of these discussions. Given the limited time physicians have in working with patients, it is unlikely that any advice given would be in-depth psychological advice. Further, it is difficult to ascertain whether these conversations were initiated by the physician or the patient. These data were unable to measure specific evidence-based obesity-related messages (limiting sugar sweetened beverages, increasing fruit and vegetables, reducing television viewing, and increasing moderate-to-vigorous physical activity) [7, 29]. Future studies should measure the impact of these areas on behavior changes. 

The potential for recall bias also exists since adolescents were asked to self-report nutrition and/or exercise data that occurred during their last physical exam, however, most adolescents (84.7%) had a physical exam at their physician visit within the past year. 

## 7. Conclusion 

This is the first study to examine trends in obesity-related counseling by race/ethnicity and by insurance type among California adolescents. Additionally, this is one of the first studies to examine trends in counseling among American Indians/Alaska Natives, a group that is also disproportionately affected by overweight and obesity [3, 4].

The findings from our study have demonstrated that the downward trend in obesity prevention counseling in California among racial/ethnic groups and health insurance groups has changed course and has begun to increase. Future analysis of the biennial CHIS surveys will indicate if the trend continues in this direction. 

It is widely accepted that obesity prevention should follow a socioecological approach, combining multiple interventions tailored to specific demographic groups [30]. While a vast amount of work has already been conducted in California to implement population-wide obesity prevention policies in schools [8] and in low-income communities [9], this momentum must also be applied to California primary health care settings. However, for physician-based counseling to continue to increase among the general adolescent population and among vulnerable high-risk groups in particular, this will require institutional and policy changes. Research on effective ways to support clinicians' use of the aforementioned evidence-based obesity-related messages is still in its infancy, although preliminary data from clinic-based obesity interventions have begun to show promising results. At least one US state (Washington) has adopted clinic-based programs to address the childhood obesity epidemic by establishing partnerships with hospitals, health care plans, and community-based organizations [31]. Other states may consider adopting such a program in the primary care setting in order to build on obesity prevention programs and policies previously implemented in schools and low-income communities. 

## Figures and Tables

**Table 1:** Characteristics of california adolescents ages 12–17, (CHIS, 2009) *n* = 3,379.

Characteristics	*n* (%)
Age ± standard deviation	14.6 ± 1.7
	range = 12–17
Gender	
Male	1767 (51.0)
Female	1612 (49.0)
Household income status	
<300% FPL	1692 (55.2)
≥300% FPL	1687 (44.8)
Race/ethnicity	
Hispanic	1226 (49.3)
American Indian/Alaska Native, not Hispanic	46 (0.7)
Asian, not Hispanic	363 (10.0)
African American, not Hispanic	100 (6.5)
White, not Hispanic	1480 (33.5)
Insurance types	
Uninsured	204 (6.3)
Low-cost/free	1055 (32.3)
Employer	1920 (56.9)
Private insurance	200 (4.5)
Weight status	
Healthy weight	2518 (71.3)
Overweight	497 (16.7)
Obese	364 (12.0)
Nutrition and/or exercise counseling	
No Discussion about nutrition or exercise	843 (24.9)
Discussion about nutrition or exercise	890 (26.9)
Discussion of both nutrition and exercise	1473 (48.2)
Last physical exam at physician visit	
Respondent never had a physical exam	38 (1.0)
≤12 months ago	2859 (84.7)
>1 year ago	482 (14.3)

Final sample size, *n* = 3,379, but may be less for some variables due to missing values.

Data presented as *n* (%), unless otherwise indicated.

FPL—Federal Poverty Level.

**Table 2:** Sample sizes for counseling variables stratified by race/ethnicity.

Race/ethnicity	2003	2005	2007	2009
Hispanic	1327	1240	1158	1164
American Indian/Alaska Native (AIAN)	41	32	21	40
Asian	237	289	246	345
African American (AA)	193	177	121	99
White	1625	1796	1657	1412

**Table 3:** Sample sizes for counseling variables stratified by insurance type.

Insurance type	2003	2005	2007	2009
Uninsured	235	210	177	172
Low-cost/free	872	893	759	1004
Employer	2359	2402	2276	1842
Private Insurance	209	251	198	188
